# Obstetric Violence in the state of Rio de Janeiro: *Nascer no Brasil II* Study

**DOI:** 10.11606/s1518-8787.2025059006536

**Published:** 2025-10-20

**Authors:** Tatiana Henriques Leite, Emanuele Souza Marques, Maria do Carmo Leal, Rosa Maria Soares Madeira Domingues, Marcos Nakamura-Pereira, Mariza Miranda Theme-Filha, Marcia Leonardi Baldisserotto, Karina de Cássia Caetano, Thaiza Dutra Gomes de Carvalho, Fernanda Freitas Fernandes, Rafaelle Mendes da Costa, Amanda Chiavazzoli, Marília Arndt Mesenburg

**Affiliations:** I Universidade do Estado do Rio de Janeiro. Instituto de Medicina Social Hesio Cordeiro. Departamento de Epidemiologia. Rio de Janeiro, RJ, Brasil; II Fundação Oswaldo Cruz. Escola Nacional de Saúde Pública Sérgio Arouca. Departamento de Métodos Quantitativos e Estatística. Rio de Janeiro, RJ, Brasil; III Fundação Oswaldo Cruz. Escola Nacional de Infectologia Evandro Chagas. Laboratório de Pesquisa Clínica em DST/Aids. Rio de Janeiro, RJ, Brasil; IV Fundação Oswaldo Cruz. Instituto Fernandes Figueira. Departamento de Ginecologia e Obstetrícia. Rio de Janeiro, RJ, Brasil; V Universidade Federal do Rio de Janeiro. Instituto de Psicologia. Departamento de Psicometria. Rio de Janeiro, RJ, Brasil

**Keywords:** Obstetric Violence, Prevalence, Paturition

## Abstract

**OBJECTIVE:**

To estimate the prevalence and co-occurrence profile of obstetric violence among women hospitalized for childbirth in the state of Rio de Janeiro, as well as its distribution according to demographic, socioeconomic, and current pregnancy-related characteristics.

**METHODS:**

This is a perinatal cohort study. Data were obtained from the second telephone follow-up of the *Nascer no Brasil II* Study referring to the state of Rio de Janeiro. Obstetric violence (physical abuse; psychological abuse; neglect; stigma and discrimination; and inappropriate vaginal examinations) was assessed using a culturally adapted version of a World Health Organization-endorsed questionnaire. Prevalence and co-occurrence profile (Venn diagram) of the types of obstetric violence were estimated.

**RESULTS:**

The overall prevalence of obstetric violence was 65.3%. The most prevalent types were inappropriate vaginal examinations (46.2%), neglect (31.5%), and psychological abuse (21.7%). Socioeconomic factors such as low educational level, unemployment, receipt of government benefits, and delivery under public healthcare financing were more frequent among women who experienced various types of obstetric violence. Other characteristics such as age ≥ 35 years, having gone into labor, and being a primipara were also associated with higher prevalence. The co-occurrence of the four most common types of obstetric violence (psychological abuse, neglect, stigma and discrimination, and inappropriate vaginal examinations) was 3%.

**CONCLUSION:**

Findings indicate that obstetric violence is a significant public health issue in the state of Rio de Janeiro, and its occurrence reflects inequalities in care for specific subgroups of postpartum women.

## INTRODUCTION

Obstetric violence was officially recognized as a significant public health issue by the World Health Organization (WHO)^1^ and the United Nations^2^ only in the 2010s. This delayed recognition may be partially attributed to prior improvements in maternal and infant morbidity and mortality indicators, which created the conditions for broader discussions on reproductive health to emerge^3^. Within this context, ensuring respectful and dignified treatment of women during abortion, pregnancy, childbirth, and the puerperium gained visibility. The prevention of obstetric violence became a recognized objective, as reflected in the Sustainable Development Goals, specifically targets 5.1 and 5.2^4^.

The term *obstetric violence* was coined by civil society movements advocating for women's reproductive rights and was first incorporated into legislation in Venezuela in 2007. Since then, the term has been adopted by other countries and is now widely used and disseminated globally. Although there is no universal consensus on its nomenclature or precise definition^5^, obstetric violence is generally understood to involve physical, sexual, and psychological abuse, negligence, unnecessary or non-evidence-based medical interventions, lack of information or informed consent for procedures, denial of legally guaranteed rights, and systemic limitations related to supplies and human resources in healthcare settings^5,6^. There is broad agreement that such practices constitute violations of human rights, including the rights to health, physical integrity, and reproductive autonomy^2^.

Obstetric violence presents specific characteristics: it occurs during the reproductive cycle, including pregnancy, childbirth, the puerperium, and abortion; it takes place within healthcare settings such as outpatient clinics, private practices, and maternity wards/hospitals; it is most often perpetrated by health professionals (although not exclusively); and it results in adverse outcomes for the health of both the woman and her newborn^7–9^.

In Brazil, the prevalence of obstetric violence ranges from 18 to 44%, according to national studies^10^, conducted in both hospital^8^ and community^11^ settings. This wide variation is attributed to several factors, including the geographic location of the study, the postpartum period during which data were collected, and the methodology used to estimate prevalence (whether point-in-time or lifetime). One of the primary limitations, however, lies in the use of different questionnaires and survey items to assess the issue, which affects both the accuracy of prevalence estimates and the comparability of findings across studies.

In 2021, the city of Rio de Janeiro launched a hotline for reporting obstetric violence, accessible via the number 1746^12^. Nevertheless, reports of obstetric violence have been frequent throughout the state, submitted through various channels, including telephone, media, and social networks. These reports have included high-profile cases, such as the 2023 incident in which a woman was raped by an anesthesiologist during a cesarean section. Despite these occurrences, no representative epidemiological studies on obstetric violence have been conducted in the state, highlighting a significant gap in the literature.

To address some of the aforementioned limitations, the *Nascer no Brasil II* Survey was designed to estimate the prevalence of obstetric violence among women hospitalized for childbirth in the state of Rio de Janeiro, to assess its prevalence within vulnerable subgroups, and to examine the co-occurrence patterns of different types of obstetric violence in this population.

## METHODS

### Study Design

This study is a hospital-based perinatal cohort with national representation. The cohort included a baseline assessment and two telephone follow-ups conducted during the second and fourth months postpartum or post-abortion. The research protocol has been previously published, with detailed information available on the baseline^13^ and follow-up procedures^14^.

### Study Population

The study population of the *Nascer no Brasil II* Survey comprised all women hospitalized for childbirth (live birth or stillbirth) or experiencing abortion in selected maternity hospitals across Brazil that reported 100 or more live births, according to the 2017 Live Birth Information System. Exclusion criteria included women with triplet or higher-order pregnancies, those with severe mental disorders, non-Portuguese-speaking foreigners, and deaf women.

Inclusion criteria for this study were women residing in the state of Rio de Janeiro who were hospitalized for childbirth. Women were excluded if they experienced stillbirths with a birth weight of less than 500 grams or a gestational age of less than 20 weeks, as these cases were classified as miscarriages.

### Sampling

The sample of postpartum women from the state of Rio de Janeiro was designed to be representative of the state's territory. A total of 29 maternity hospitals were included: 14 public, 12 private, and 3 mixed. The sample size was calculated based on the 2019 cesarean section rate in the state (57%), with a significance level of 5% and 90% power to detect differences of 7%. A design effect of 1.3 was applied, resulting in a minimum required sample size of 1,350 postpartum women. Post-hoc calculations indicated that the sample had sufficient power to detect absolute differences of 5% for outcomes with prevalence rates between 5 and 20%.

### Data Source

This study utilized data from hospital interviews conducted with women after childbirth (sociodemographic information, telephone contact details, and data on prenatal and hospital care) and from the second telephone follow-up questionnaire, which focused on obstetric violence.

### Data Collection Procedure

Women were approached in their hospital beds, either in individual rooms or shared wards, at least six hours after childbirth by a trained interviewer. At that time, they were invited to participate in the study, which consisted of three interviews: the first conducted in the hospital (baseline) and two subsequent follow-ups by telephone. Consent was also requested to collect information from their prenatal cards and medical records.

### Variables and Measurement Instruments

Demographic, socioeconomic, and current pregnancy variables were obtained from the hospital questionnaire. For demographic characteristics, women's age (in years) was categorized into three groups: adolescents (≤19 years), adults (20–34 years), and adults with higher obstetric risk (35 years old or older). Self-reported skin color was recorded according to the Brazilian Institute of Geography and Statistics classification, which includes five categories: white, black, brown, Asian, and Indigenous. However, due to the low number of women identifying as Indigenous (5) or Asian (3), these responses were excluded from the analysis, resulting in three final categories: white, black, and brown. Educational attainment was categorized based on the highest grade completed: up to 8 years of schooling (incomplete elementary education), 9–11 years (incomplete secondary education), and ≥12 years (complete secondary education). Marital status was classified as living with a partner or not.

For socioeconomic characteristics, women were asked whether they had paid employment during pregnancy (yes or no) and whether they or anyone in their household received *Bolsa Família* or any other government social benefit, including emergency aid provided during the COVID-19 pandemic (yes or no).

Regarding the current pregnancy, parity was categorized as primiparous or multiparous. Women were also asked about the adequacy of prenatal care, with eight or more consultations during pregnancy considered adequate^15^. Additional information included whether the woman went into labor at the hospital/maternity ward where the birth occurred (yes or no), the duration of labor (< 2 hours, 2–8 hours, or ≥ 8 hours), the continuous presence of a companion (yes or no), and whether a doula was present during labor or delivery (yes or no). Mode of delivery was recorded as vaginal (including forceps or vacuum-assisted) or cesarean section. The source of delivery financing was classified as public or private. During the hospital interview, women self-reported their height and pre-pregnancy weight, which were used to calculate pre-gestational body mass index, categorized as eutrophic, overweight, or obese.

The obstetric violence questionnaire was administered during the second telephone follow-up, conducted four months after delivery. A cross-culturally adapted version of the instrument proposed by Bohren et al.^6^ was used. The questionnaire comprises five dimensions: physical abuse (7 items); psychological abuse (13 items); neglect (4 items); stigma and discrimination (6 items); and inappropriate vaginal touching (5 items), totaling 35 items.

To estimate the prevalence within each dimension, obstetric violence was considered present if the respondent gave a positive response to at least one item within that dimension. For the overall prevalence of obstetric violence, a positive response to at least one item across any of the five dimensions was considered indicative of its occurrence.

### Statistical Analysis

All analyses accounted for baseline sampling weights, as well as follow-up sampling weights calibrated according to hospital and maternal characteristics associated with response to the second telephone interview. This calibration aimed to minimize the impact of follow-up losses. A logistic regression model was used to estimate the probability of each woman responding to the follow-up interview, employing propensity scores based on variables that distinguished respondents from non-respondents. This approach sought to adjust for response bias by assuming that non-respondents would, on average, have answered similarly to respondents within each stratum and adjustment category. Variables related to place of birth, sociodemographic characteristics, and obstetric factors were tested. The final, most parsimonious model included only those variables that were statistically significant (p < 0.05) in differentiating respondents from non-respondents. Non-respondents were more likely to have less than 16 years of education, to have delivered in public hospitals (compared to private or mixed hospitals), and in facilities with fewer than 500 births/year. The primary reasons for non-response were incorrect telephone numbers and failure to answer calls.

A descriptive analysis was then conducted to examine the demographic, socioeconomic, and current pregnancy characteristics of the study population. The prevalence of different types of obstetric violence was analyzed across subgroups based on these characteristics. The χ² test was used to assess the homogeneity of prevalence among the subgroups, with a p-value < 0.05 considered indicative of statistically significant differences.

The co-occurrence profile of obstetric violence was analyzed using two approaches. The first involved counting the number of different types of obstetric violence experienced, with prevalence assessed across subgroups. The second approach consisted of a graphical representation of co-occurrence using a Venn diagram, illustrating the overlap among the four most frequent types of obstetric violence.

All analyses were conducted using Stata software, version 17.0, employing the *svy* command to account for the complex sampling design.

### Ethical Aspects

This study was approved by the National Research Ethics Committee (approval No. 3.909.299) and by local research ethics committees, when required by the selected hospitals. Prior to each interview, participants were asked to provide consent after reading the Informed Consent Form. For minors, an assent form was used.

## RESULTS

A total of 1,923 postpartum women were interviewed at the research baseline. After excluding 120 cases of abortion, the final sample comprised 1,881 postpartum women. The second telephone follow-up interviewed 1,190 women, representing 67.5% of the baseline sample. Most of the interviewees (72.7%; 95% confidence interval — 95%CI 69.8–75.3) were between 20 and 34 years old. The majority self-identified as brown (44.4%; 95%CI 39.3–49.6) and had completed high school or higher education (56.6%; 95%CI 45.7–66.9). Regarding marital status, 88.6% (95%CI 86.3–90.5) reported having a partner; 52.5% (95%CI 48.9–56.2) did not work throughout the entire pregnancy; and 57.3% (95%CI 34.5–51.4) received some form of social benefit from the government, such as *Bolsa Família* and/or *Auxílio Brasil*. Regarding pregestational nutritional status, 30.7% (95%CI 26.2–35.6) of the women were overweight, and 25.5% (95%CI 21.4–35.6) were obese. Most women were multiparous (63.2%; 95%CI 60.5–65.9) and received adequate prenatal care (78.9%; 95%CI 75.7–81.7) (data not shown in a table).

Regarding the peripartum and delivery period, 44.8% (95%CI 38.5–51.2) of women went into labor, with most experiencing labor lasting between two and eight hours (43.3%; 95%CI 39.3–47.3). Almost all women had a companion present throughout (95.0%; 95%CI 85.4–98.4); however, only a small proportion had a doula present during labor or delivery (2.9%; 95%CI 1.9–4.4). The majority of deliveries were by cesarean section (59.8%; 95%CI: 52.1–67.0), and most births were publicly funded (73.7%; 95%CI 60.3–83.8) (data not shown in a table).

Table 1 presents the frequency of each act of violence experienced, as well as the prevalence by type of obstetric violence and the overall prevalence of violence. The most prevalent types of obstetric violence were inappropriate vaginal touching (46.2%), followed by neglect (31.5%), psychological abuse (21.7%), stigma and discrimination (7.8%), and physical abuse (3.1%). The overall prevalence of obstetric violence was 65.3%. Regarding the perpetrator of the violence, nursing professionals were most frequently cited by the women interviewed. In terms of timing, the majority of incidents of obstetric violence occurred during the peripartum/partum period.

**Table 1:** Prevalence by type of violence experienced, nature of the violence, and total among postpartum women residing in the state of Rio de Janeiro, RJ, 2022/2023, *Estudo Nascer no Brasil 2* (n = 1,761).

		Total sample		
		n	%	95%CI
**Acts of physical abuse experienced**				
	Pinching	-	-	-
	Being slapped or punched	-	-	-
	Being kicked	-	-	-
	Being hit with an object	1	0.08	0.01–6.62
	Being gagged or having a hand placed over the mouth to prevent speaking or making noise	-	-	-
	Being forcibly restrained, choked, or tied to the bed	4	0.38	0.11–1.28
	Having the belly squeezed or someone pressing down on it to "help" the baby come out	53	2.68	2.00–3.61
	Experienced at least one act of physical abuse	58	3.14	2.39–4.12
**Professional category of the perpetrator of the physical abuse** a				
	Physician	1	21.73	0.09–98.8
	Nursing staff	2	45.64	19.67–85.50
	Other professionals	1	15.71	0.23–93.87
	Unknown	1	16.92	0.06–98.45
**When the physical abuse occurred**				
	Pre-delivery/delivery	11	65.59	47.50–80.07
	Postpartum	5	29.71	15.55–49.23
	Both moments	2	4.70	0.41–37.17
**Acts of psychological abuse experienced**				
	Yelling or shouting	49	4.61	3.68–5.76
	Offending or insulting	41	3.12	1.71–5.63
	Scolding or reprimanding	121	10.87	9.34–12.63
	Mocking	85	6.85	4.57–10.15
	Negative comments about physical appearance	18	1.46	0.63–3.32
	Negative comments about the baby's appearance	6	0.47	0.19–1.17
	Negative comments about sexual life	39	3.70	2.83–4.84
	Threatening to perform a medical procedure against your will	37	3.78	2.57–5.53
	Threatening physical abuse	1	0.13	0.02–1.07
	Threatening that, if not obeyed, the woman or baby would have problems	32	2.96	2.28–3.82
	Threatening to withhold or stop care for the woman or baby	14	1.18	0.07–2.06
	Blaming the woman for something that happened to her or the baby	40	3.93	2.97–5.19
	Sighing or grumbling	106	10.10	8.59–11.84
	Experienced at least one act of psychological abuse	209	21.68	17.16–27.00
**Professional category of the perpetrator of psychological abuse**				
	Physician	52	23.31	18.76–28.57
	Nursing staff	108	54.35	46.37–62.12
	Other professionals	25	10.90	6.07–18.81
	Unknown	21	11.44	8.31–15.54
**When the psychological abuse occurred**				
	Pre-delivery/delivery	102	48.59	38.26–59.03
	Postpartum	77	38.97	27.17–52.22
	Both moments	25	12.45	6.78–21.75
**Acts of neglect experienced**				
	Feeling ignored by healthcare professionals and other staff	207	19.95	17.63–22.49
	Feeling abandoned by healthcare professionals and other staff	168	17.01	14.14–20.32
	Feeling like your presence was a nuisance	84	8.29	6.92–9.89
	Having to wait long periods before being attended to	250	22.2	19.21–25.50
	Experienced at least one act of neglect	908	31.45	29.11–33.89
**Acts related to stigma and discrimination experienced**				
	Receiving negative comments about race/skin color	2	0.48	0.13–1.75
	Receiving negative comments about religion	5	0.37	0.12–1.09
	Receiving negative comments about age	56	4.78	3.68–6.18
	Receiving negative comments about having/not having a partner	18	1.81	1.05–3.09
	Receiving negative comments about education or financial situation	6	0.39	0.14–1.08
	Receiving negative comments about health condition	18	1.77	0.70–4.40
	Experienced at least one act of stigma and discrimination	81	7.79	6.25–9.66
**Professional category of the perpetrator of stigma and discrimination**				
	Physician	14	19.99	13.22–29.07
	Nursing staff	38	43.58	32.63–55.20
	Other professionals	11	16.06	9.87–24.99
	Unknown	18	20.37	14.22–28.30
**When the act of stigma and discrimination occurred**				
	Pre-delivery/delivery	32	45.16	32.23–58.77
	Postpartum	23	32.80	22.09–45.67
	Both moments	14	22.04	9.87–42.20
**Acts related to inappropriate vaginal exams**				
	No explanation was given for why the vaginal exam was necessary	363	35.24	29.50–41.45
	Did not ask for your permission before performing the vaginal exam	226	22.48	17.66–28.18
	Confidential health information was spoken aloud so others could hear	56	5.34	3.78–7.51
	Vaginal exams were not conducted privately/with privacy	221	19.81	16.60–23.47
**Experienced at least one inappropriate vaginal exam**		435	46.23	39.46–53.15
**Experienced at least one type of obstetric violence**		1,219	65.30	58.60–71.44

95%CI: 95% confidence interval.

a The question regarding the perpetrator of the act of physical abuse was asked during the second telephone interview, in which the question regarding the occurrence of the Kristeller maneuver (*i.e*., pressing on the abdomen or climbing onto it to "help" the baby come out) was not included. This item was part of the hospital-based (baseline) questionnaire.

Table 2 presents the prevalence of types of violence according to the characteristics of the women interviewed. The overall prevalence of obstetric violence was higher among women who were not employed and those whose childbirth was publicly funded. Women who received some form of government assistance also had a higher prevalence of obstetric violence across all dimensions, except for physical abuse. Women over 35 years of age, with less than 12 years of education, and those who experienced labor, were more likely to experience obstetric violence in three different forms (neglect; stigma and discrimination; and inappropriate vaginal examinations). Primiparous women and those with labor lasting longer than eight hours also reported higher prevalences of obstetric violence in two dimensions (psychological abuse and neglect). Finally, black women, those without a partner, and those with inadequate prenatal care had a higher prevalence of stigma and discrimination.

**Table 2:** Prevalence of obstetric violence by type according to demographic, socioeconomic, and current pregnancy characteristics of postpartum women residing in the state of Rio de Janeiro, RJ, 2022/2023, *Estudo Nascer no Brasil 2* (n = 1,761).

		Physical abuse		Psychological abuse		Negligence		Stigma and discrimination		Inappropriate vaginal examinations	
		%	95%CI	%	95%CI	%	95%CI	%	95%CI	%	95%CI
**Age (years)**											
	**≤** 19	3.13	2.35–4.16^a^	24.40	16.88–33.90	32.28	28.82–35.95	7.89	5.99–10.33^b^	45.97	40.16–51.90^a^
	20–34	1.21	0.26–5.38	8.65	4.62–15.64	26.87	20.57–34.26	2.01	0.83–4.77	40.23	30.11–51.25
	**≥** 35	6.46	3.59–11.37	24.01	11.82–42.71	33.17	20.30–49.17	16.54	11.30–23.56	58.99	47.66–69.45
**Race/skin color**											
	White	3.75	2.39–5.84	18.44	10.71–29.89	30.07	25.51–35.06	5.61	3.48–8.96^a^	39.43	28.27–51.81
	Black	0.85	0.18–4.05	24.75	18.03–32.98	29.25	25.03–33.86	10.95	8.61–13.82	51.73	39.66–63.60
	Brown	4.05	2.29–7.05	22.07	18.75– 25.79	33.63	29.53–38.00	7.44	5.38–10.21	47.45	42.36–52.61
**Education (years of study)**											
	> 12	2.11	1.30–3.42	18.57	14.54–23.40^a^	29.44	23.23–32.86	4.78	3.21–7.05^a^	37.69	32.98–42.65^a^
	8–12	4.52	3.26–6.25	26.95	23.03–31.27	34.25	25.22–44.59	10.56	7.35–14.95	58.72	50.32–66.64
	< 8	4.43	1.64–11.41	22.86	15.24–32.81	33.86	25.62–43.20	14.19	7.90–24.15	53.67	44.18–62.91
**Marital status**											
	Without partner	5.39	2.85–9.97	22.42	16.90–29.12	37.86	30.56–45.75	14.61	9.31–22.18^a^	43.46	34.43–53.14
	With partner	2.86	2.14–3.82	21.49	16.67–27.25	30.56	28.19–33.04	6.92	5.33–8.95	46.46	39.40–53.66
**Paid work during pregnancy**											
	No	4.25	3.09–5.83^a^	24.24	19.25–30.03^a^	33.84	31.19–36.61^a^	10.72	8.78–13.02^b^	53.45	43.27–63.35^a^
	Yes	1.92	1.20–3.04	18.85	14.21–24.57	28.80	25.31–32.57	4.55	3.07–6.69	38.21	33.23–43.45
**Received *Bolsa Família*, social benefit, or emergency government aid**											
	No	3.09	1.61–5.86	16.21	11.90–21.68^a^	25.94	22.73–29.42^b^	3.61	2.02–6.38^a^	40.70	32.77–49.14^a^
	Yes	3.19	2.25–4.51	25.85	20.26–32.35	35.59	32.26–39.08	10.91	8.69–13.61	50.13	44.05–56.20
**Parity**											
	Primiparous	4.57	3.14–6.61^a^	26.49	20.43–33.60^b^	35.15	30.12–40.53	7.77	5.19–11.49	45.54	41.28–49.87
	Multiparous	2.32	1.44–3.71	18.8	14.89–23.42	29.33	25.62–33.34	7.81	5.99–10.12	46.68	37.79–55.78
**Adequate prenatal care**											
	No	6.89	4.28–10.89	21.79	12.73–34.75	37.15	29.70–45.26	15.29	11.21–20.52^b^	50.76	46.20–55.31
	Yes	2.32	1.52–3.53^a^	21.92	14.60–31.57	30.41	27.86–33.09	5.83	4.41–7.66	44.89	36.90–53.15
**Went into labor**											
	No	0.83	0.22–3.03^a^	19.01	12.05–28.67	27.06	22.57–32.08^a^	7.06	5.08–9.74	42.67	36.14–49.45^a^
	Yes	6.08	4.13–8.85	24.93	21.57–28.64	36.93	33.14–40.90	8.62	6.50–11.34	48.88	41.44–56.36
**Labor duration (hours)**											
	< 2	0.00	-	20.37	12.33–31.75^a^	29.39	23.70–35.81^b^	9.13	5.45–14.92	50.02	38.55–61.48
	2 to 8	3.57	1.54–8.03	15.71	12.46–19.61	22.44	18.49–26.96	5.80	3.99–8.36	42.06	32.99–51.70
	**≥** 8	4.37	2.12–8.80	27.60	24.82–30.57	40.79	28.41–43.50	8.60	6.40–11.45	50.43	43.84–57.01
**Delivery mode**											
	Vaginal	7.11	5.55–9.06^b^	23.70	21.14–26.47	33.18	27.70–39.17	7.42	5.38–10.17	50.68	40.92–60.40
	Cesarean	0.48	0.14–1.64	20.32	13.46–29.48	30.29	25.53–35.51	8.04	5.63–11.36	42.88	37.54–48.40
**Companion present at all times**											
	No	3.77	1.11–12.08	18.82	5.59–47.56	35.67	28.91–42.76	6.96	5.34–9.02	56.44	43.92–68.20
	Yes	2.63	1.88–3.65	21.05	15.65–27.69	30.67	28.48–32.94	7.84	2.86–19.75	44.44	35.86–53.86
**Doula present**											
	No	2.79	1.96–3.97	21.55	16.41–27.75	32.26	29.33–35.33	7.65	5.90–9.86	45.21	39.29–51.27
	Yes	11.14	2.62–36.86	26.32	14.39–43.17	13.56	4.51–34.25	2.33	0.30–15.97	56.36	30.75–78.98
**Funding**											
	Public	4.04	3.08–5.26^b^	26.66	23.01–30.65^b^	35.03	32.57–38.13^b^	10.06^a^	8.36–12.05^b^	51.39	45.02–57.72^b^
	Private	0.65	0.21–2.00	7.43	4.00–13.38	20.64	16.08–26.09	1.33	0.55–3.16	29.39	22.82–36.96
**Pre-gestational BMI**											
	Normal weight	3.08	1.55–6.02	23.83	13.69–38.15	34.60	27.45–42.51	7.75	5.64–10.56	44.51	32.53–57.16
	Overweight	3.62	1.79–7.17	16.01	11.04–22.64	27.75	23.06–32.98	5.02	2.50–9.82	42.44	32.71–52.80
	Obesity	1.92	0.47–7.52	22.01	17.08–27.89	31.85	26.34–37.91	7.04	4.36–11.18	43.17	33.73–53.13

95%CI: 95% confidence interval; BMI: body mass index.

a p ≤ 0.05;

b p ≤ 0.01.

The accumulation of different forms of obstetric violence, based on various characteristics of the women interviewed, is presented in Table 3. It was found that approximately one-third of the postpartum women reported experiencing at least one type of obstetric violence. Notably, women over 35 years of age and adolescents, those with less than 12 years of schooling, those without paid work and/or receiving government benefits, primiparous women, those who went into labor, those with prolonged labor, those who had vaginal deliveries, and those who received publicly funded childbirth assistance experienced a greater number of forms of obstetric violence compared to women without these characteristics.

**Table 3:** Accumulation of different forms of obstetric violence experienced according to demographic, socioeconomic, and current pregnancy-related characteristics of postpartum women residing in the state of Rio de Janeiro, RJ, 2022/2023, *Estudo Nascer no Brasil 2* (n = 1,761).

		0 OV		1 OV		2 OV		+3 OV		p-value
		%	95%CI	%	95%CI	%	95%CI	%	95%CI	
**Total sample**		38.03	30.51–46.18	32.56	28.44–36.96	16.64	13.73–20.01	12.77	10.43–15.54	
**Age (years)**										
	**≤** 19	39.10	32.41–46.23	27.82	24.50–31.41	19.02	14.71–24.22	14.06	10.31–18.88	
	20–34	43.57	29.41–58.86	45.42	27.86–64.21	6.26	2.89–13.03	4.75	2.26–9.68	0.0204
	**≥** 35	19.60	13.43–27.68	45.54	27.84–64.44	17.29	10.40–27.35	17.57	9.85–29.37	
**Race/skin color** a										
	White	42.37	29.89–55.92	32.40	25.17–40.58	15.14	8.92–24.55	10.08	7.69–13.11	
	Black	35.94	28.48–44.15	33.91	28.62–39.65	14.45	10.90–18.90	15.70	10.37–23.06	0.3157
	Brown	36.41	30.67–42.56	31.87	27.24–36.88	18.90	16.11–22.04	12.83	9.82–16.59	
**Education (years of study)**										
	> 12	44.22	37.00–51.69	33.84	7.64–40.64	12.50	10.21–15.21	9.45	7.45–11.91	
	8–12	28.06	22.34–34.6	29.18	24.21–34.71	24.40	19.95–29.47	18.35	14.77–22.58	0.0004
	< 8	34.97	23.98–47.82	34.33	27.03–42.45	16.58	10.55–25.08	14.12	6.73–27.26	
**Marital status**										
	Without partner	38.84	31.36–46.88	32.85	28.74–37.24	15.89	13.02–19.26	12.43	9.79–15.65	
	With partner	32.51	21.88–45.32	30.33	20.9–41.77	22.70	17.73–28.57	14.46	10.19–20.11	0.1466
**Paid work during pregnancy**										
	No	30.67	19.68–44.41	36.83	27.41–47.38	16.78	14.57–19.25	15.72	12.16–20.08	
	Yes	46.07	41.52–50.69	27.89	22.17–34.45	16.48	10.81–24.32	9.55	7.80–11.66	0.0654
**Received *Bolsa Família*, another social benefit, or emergency government aid**										
	No	43.82 35.24–52.78		33.41 27.03–40.47 13.72			9.00–20.36	9.05	6.48–12.51	
	Yes	33.92	26.78–41.87	31.87	27.39–36.71	18.76	16.31–21.47	15.46	11.57–20.35	0.0232
**Parity**										
	Primiparous	37.53	30.10–45.61	27.29	22.09–33.19	16.41	12.48–21.28	18.77	13.60–25.32	
	Multiparous	38.36	29.88–47.62	35.47	29.12–42.37	16.78	13.68–20.42	9.39	8.08–10.88	0.0046
**Adequate prenatal care**										
	Yes	33.15	27.52–39.3	31.91	25.21–39.45	23.05	18.36–28.51	11.89	6.75–20.12	
	No	39.03	29.91–48.99	32.84	28.31–37.72	14.80	11.23–19.27	13.33	10.16–17.30	0.1951
**Went into labor**										
	No	43.36	34.95–52.16	32.99	27.95–38.47	12.88	9.38–17.43	10.77	6.78–16.68	
	Yes	32.99	25.91–40.93	31.38	27.92–35.07	20.63	16.71–25.20	14.99	12.05–18.51	0.0153
**Labor duration (hours)**										
	< 2	36.37	23.85–51.06	33.24	26.57–40.66	17.18	12.55–23.07	13.2	9.51–18.05	
	2 to 8	44.41	34.55–54.74	35.19	34.55–54.74	14.41	8.56–23.24	5.99	3.94–9.01	0.0077
	**≥** 8	32.48	28.08–37.22	28.63	24.36–33.33	19.93	15.52–25.24	18.95	15.29–23.24	
**Delivery mode**										
	Vaginal	33.72	27.85–40.14	32.00	27.32–37.07	22.57	19.28–26.24	11.71	9.27–14.67	
	Cesarean	41.28	32.19–51.01	32.97	28.34–37.96	12.18	8.73–16.75	13.57	9.11–19.72	0.0149
**Companion present at all times**										
	No	38.12 28.40–48.90		26.22	15.21–41.33	19.08	10.90–31.25	16.57	5.86–38.82	
	Yes	38.80	30.01–48.37	32.57	28.03–37.46	16.81	13.88–20.22	11.82	8.77–15.73	0.6822
**Doula present**										
	No	38.86	31.43–46.86	31.87	27.76–36.29	16.05	13.30–19.25	13.21	10.33–16.74	
	Yes	38.40	18.36–63.33	22.08	8.14–47.53	31.05	8.73–67.94	8.48	2.31–26.63	0.3773
**Funding**										
	Public	32.55	25.66–40.28	31.83	27.20–36.86	20.06	17.89–22.42	15.56	13.63–17.71	0.0000
	Private	55.78	47.39–63.86	34.90	25.14–46.12	5.58	3.07–9.94	3.74	1.73–7.88	
**Pre-gestational body mass index**										
	Normal weight	39.77	27.81–53.10	26.56	22.28–31.33	19.26	12.88–27.77	14.41	8.44–23.52	
	Overweight	41.37	33.37–49.85	36.55	28.32–45.65	11.69	7.01–18.85	10.39	6.66–15.86	0.2972
	Obesity	43.85	33.49–54.77	28.28	17.98–41.49	12.16	7.69–18.70	15.71	10.80–22.31	

OV: Obstetric violence; 95%CI: 95% confidence interval.

**Figure 1:**
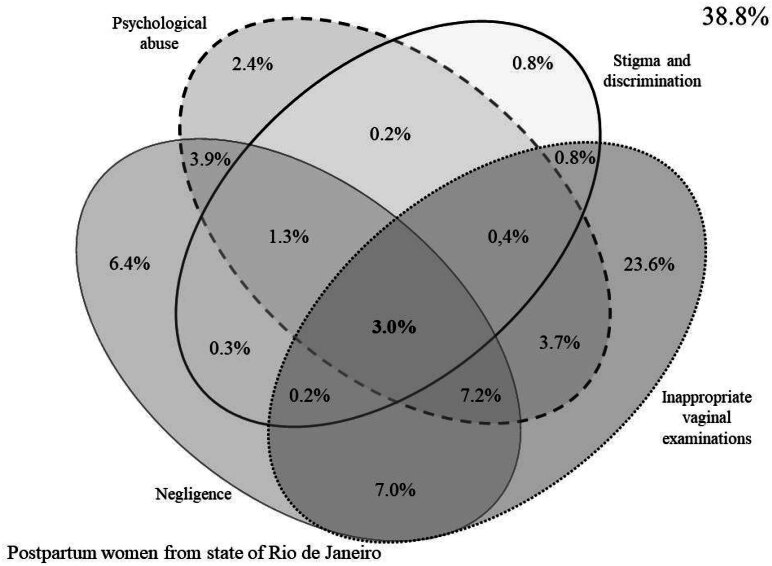
Venn Diagram: postpartum women from state of Rio de Janeiro.

The co-occurrence profile of obstetric violence among postpartum women in the state of Rio de Janeiro is shown graphically in Figure 1. It can be observed that 38% of the sample reported not experiencing any of the most prevalent types of obstetric violence in the state. The co-occurrence of the four most prevalent types of obstetric violence (psychological abuse, neglect, stigma and discrimination, and inappropriate vaginal touching) was reported by 3% of the participants.

## DISCUSSION

The findings of this study indicate that obstetric violence is a prevalent issue in maternity hospitals in the state of Rio de Janeiro. Approximately two-thirds of the women interviewed reported experiencing at least one type of violence investigated, with inappropriate vaginal examinations and neglect being the most commonly reported forms. The pre-labor and labor periods were identified as the times when obstetric violence occurred most frequently, with nursing professionals being the primary perpetrators. The results also highlighted disparities in the occurrence of this issue, with women in more vulnerable socioeconomic and demographic conditions experiencing a higher prevalence of obstetric violence. Additionally, certain physiological characteristics of labor were identified as markers for the occurrence of obstetric violence.

When comparing our findings to those of the study by Bohren et al.^16^, a multicenter study conducted in four African countries (Ghana, Guinea, Myanmar, and Nigeria), the prevalence of nonconsensual vaginal touching was found to be 59.4%, which is higher than the rate observed in the present study. Conversely, neglect was reported by only 4.5% of women in the African study, significantly lower than the prevalence found in our study. For other types of violence (physical abuse, psychological abuse, stigma, and discrimination), Bohren et al.^16^ reported average prevalences of 14, 37.8, and 0.6%, respectively. The prevalence of physical abuse was four times higher in the African countries, while psychological abuse and stigma and discrimination were more frequently reported in the state of Rio de Janeiro, with age discrimination being the most common form. These discrepancies can likely be attributed to cultural and socioeconomic differences, variations in local health services, differences in the current health management models, and the varying perceptions of obstetric violence among women in these different settings.

Regarding prevalence by demographic and socioeconomic characteristics, it is noteworthy that obstetric violence was more frequently reported by women who were adolescents or aged over 35 years, lacked paid work during pregnancy, received government assistance, or had publicly funded childbirth. These women exhibited a higher prevalence of nearly all types of obstetric violence, with the exception of physical abuse. A similar pattern was observed in the accumulation of different forms of violence, with the experience of three or more types being more common among women in more vulnerable socioeconomic conditions. These findings suggest that obstetric violence is not randomly distributed but disproportionately affects poorer women, highlighting structural inequalities in the provision of healthcare and violating the principle of equality established by the Brazilian Federal Constitution^17^ and reaffirmed by the Convention of Belém do Pará^18^.

With regard to current pregnancy characteristics, being a primiparous woman, experiencing labor, and having prolonged labor were the main markers associated with obstetric violence. These findings underscore the insufficient preparedness of healthcare teams to provide appropriate monitoring and emotional support during labor, which, instead, contribute to the occurrence of obstetric violence. Supporting these results, it was also observed that all types of obstetric violence predominantly occurred during the pre-labor and labor periods^16^. These are times of intensified interaction between the woman and healthcare professionals, as well as periods of heightened emotional tension for all parties involved. Similar patterns have been reported in other countries, as demonstrated in the study by Bohren et al.^16^.

A common factor across all forms of obstetric violence analyzed was the identity of the perpetrator. The women interviewed most frequently reported nursing professionals as the main individuals responsible for the acts of violence they experienced. Although this may appear paradoxical, given that nursing is one of the primary proponents of the childbirth humanization movement, similar findings have been reported in studies conducted in Latin America and other regions of the world^16,19^. This can be attributed to the fact that nursing professionals are responsible for providing continuous care and tend to spend more time in close contact with women, particularly during labor, which is both the most emotionally intense phase and the period in which obstetric violence most frequently occurs. Poor working conditions, including staff shortages and excessive workloads, may lead to physical and emotional exhaustion, contributing to the behaviors most frequently reported by women, such as "huffing and puffing," "reprimanding or scolding," and "feeling ignored or abandoned by professionals." It should also be noted that the nursing category includes professionals with mid- and high-level education. Thus, mitigating obstetric violence involves ensuring adequate working conditions and training and valuing nursing professionals.

Specific patterns were observed depending on the type of obstetric violence analyzed. Physical violence presented a low overall prevalence, predominantly associated with the Kristeller maneuver. However, according to the Live Birth Information System, approximately 180,000 children were born in the state of Rio de Janeiro in 2022. Applying the observed prevalence (3.14%) suggests that roughly 5,600 women experienced one of the most severe forms of physical violence during childbirth. Notably, almost all cases of physical violence occurred among women who had publicly funded vaginal births. A similar trend was observed for stigma and discrimination, which were also predominantly reported in publicly financed deliveries. One possible explanation for these findings lies in the care model adopted in privately funded births, in which prenatal and childbirth care are typically provided by the same professional, potentially reducing the incidence of stigma and discrimination. Additionally, the higher frequency of cesarean sections among women with private financing may account for the lower occurrence of the Kristeller maneuver. Nevertheless, it is important to emphasize that even among women with privately financed births, the prevalence of inappropriate vaginal examinations and neglect exceeded 20%. These findings indicate that while women with publicly financed births are more severely affected by obstetric violence, women with privately financed births are not exempt from such experiences.

The findings of this study should be interpreted in light of its strengths and limitations. Among its strengths is the use of a representative sample from the state of Rio de Janeiro, particularly significant given the absence of state-level studies on obstetric violence in Brazil. Furthermore, the use of a specific instrument for measuring obstetric violence, adapted from a questionnaire endorsed by the World Health Organization, underscores both the originality and methodological rigor of the research. This approach also enhances the comparability of results with those of other national and international studies.

The results presented should be interpreted in light of the study's strengths and limitations. A key strength is the use of a representative sample from the state of Rio de Janeiro, given the lack of state-level studies on obstetric violence in Brazil. Additionally, the use of a standardized and WHO-endorsed questionnaire to assess obstetric violence reinforces the originality and methodological rigor of the research, while also enhancing comparability with findings from other studies.

Regarding limitations, it is important to acknowledge the 32.5% loss to follow-up from the baseline sample, a proportion comparable to that reported in other perinatal cohort studies^20^. Previous analyses indicated that these losses were not random. Therefore, a statistical correction was applied to minimize potential underestimation of obstetric violence in the population studied. Additionally, the broad classification of the nursing team precluded differentiation between professionals with distinct levels of training, as many respondents were unable to distinguish among them.

In conclusion, obstetric violence is a frequent occurrence among women in the state of Rio de Janeiro. Unfavorable socioeconomic conditions, along with specific pregnancy and childbirth-related factors, are significant markers of its occurrence, underscoring the inequalities in the care provided to women during childbirth. Educational interventions targeting both health professionals and women, implemented within hospitals and maternity wards, may contribute to mitigating this form of violence.

It is hoped that the findings of this study will raise awareness among healthcare managers and professionals regarding the issue of obstetric violence and promote the development of public policies aimed at reducing its occurrence among parturients in the state of Rio de Janeiro, ensuring respect and dignity during this critical and unique moment in women's lives.

## Data Availability

The datasets generated and/or analyzed during the study are available from the corresponding author upon request.
