# Supplementary material for: Obstetric Violence in the state of Rio de Janeiro: Nascer no Brasil II Study
Source: Rev Saude Publica. 2025 Oct 20;59(Suppl 1):e6s. doi: 10.11606/s1518-8787.2025059006536 (PMC12539635; doi:10.11606/s1518-8787.2025059006536)
Supplement: Translation

# Translation: Violência obstétrica no estado do Rio de Janeiro: Pesquisa Nascer no Brasil II

**Authors:** Tatiana Henriques Leite, Emanuele Souza Marques, Maria do Carmo Leal, Rosa Maria Soares Madeira Domingues, Marcos Nakamura-Pereira, Mariza Miranda Theme-Filha, Marcia Leonardi Baldisserotto, Karina de Cássia Caetano, Thaiza Dutra Gomes de Carvalho, Fernanda Freitas Fernandes, Rafaelle Mendes da Costa, Amanda Chiavazzoli, Marília Arndt Mesenburg

**Affiliations:** I Universidade do Estado do Rio de Janeiro. Instituto de Medicina Social Hesio Cordeiro. Departamento de Epidemiologia. Rio de Janeiro, RJ, Brasil; II Fundação Oswaldo Cruz. Escola Nacional de Saúde Pública Sérgio Arouca. Departamento de Métodos Quantitativos e Estatística. Rio de Janeiro, RJ, Brasil; III Fundação Oswaldo Cruz. Escola Nacional de Infectologia Evandro Chagas. Laboratório de Pesquisa Clínica em DST/Aids. Rio de Janeiro, RJ, Brasil; IV Fundação Oswaldo Cruz. Instituto Fernandes Figueira. Departamento de Ginecologia e Obstetrícia. Rio de Janeiro, RJ, Brasil; V Universidade Federal do Rio de Janeiro. Instituto de Psicologia. Departamento de Psicometria. Rio de Janeiro, RJ, Brasil

**Keywords:** Violência Obstétrica, Prevalência, Parto

## Abstract

**OBJETIVO:**

Estimar a prevalência e o perfil de coocorrência de violência obstétrica em mulheres internadas por motivos de parto no estado do Rio de Janeiro, bem como sua distribuição de acordo com características demográficas, socioeconômicas e referentes à gestação atual.

**MÉTODOS:**

Trata-se de uma coorte perinatal. Os dados utilizados foram obtidos no segundo seguimento telefônico da Pesquisa Nascer no Brasil II referente ao estado do Rio de Janeiro. A violência obstétrica (abuso físico; abuso psicológico; negligência; estigma e discriminação; e toques vaginais inadequados) foi avaliada por meio de uma versão adaptada transculturalmente de um questionário endossado pela Organização Mundial da Saúde. Estimaram-se as prevalências e o perfil de coocorrência (diagrama de Venn) dos tipos de violência obstétrica analisados.

**RESULTADOS:**

A prevalência total de violência obstétrica total foi de 65,3%. Os tipos de violência obstétrica mais prevalentes foram toques vaginais inadequados (46,2%), negligência (31,5%) e abuso psicológico (21,7%). Destaca-se que características socioeconômicas como baixa escolaridade, ausência de emprego, recebimento de algum benefício governamental e ter tido o parto com financiamento público se mostraram mais presentes entre as mulheres vítimas de diferentes tipos de violência obstétrica. Outras características como idade ≥ 35 anos, entrar em trabalho de parto e ser primípara apresentaram maiores prevalências de violência obstétrica. A coocorrência dos quatro tipos de violência obstétrica mais prevalentes (abuso psicológico, negligência, estigma e discriminação e toques vaginais inadequados) foi de 3%.

**CONCLUSÃO:**

Os achados mostraram que a violência obstétrica é um problema de saúde pública relevante no estado do Rio de Janeiro, entretanto sua ocorrência evidencia desigualdade no atendimento a certos subgrupos de puérperas.

## INTRODUÇÃO

A violência obstétrica foi oficialmente reconhecida como um importante problema de saúde pública pela Organização Mundial da Saúde (OMS)^1^ e pela Organização das Nações Unidas^2^ apenas na década de 2010. Esse reconhecimento tardio pode ser parcialmente atribuído às melhorias nos indicadores de morbimortalidade materna e infantil, que possibilitaram a abertura de discussão sobre outras questões relacionadas à saúde reprodutiva^3^. Nesse contexto, o tratamento respeitoso e digno das mulheres durante o atendimento a aborto, gravidez, parto e puerpério e o enfrentamento à violência obstétrica ganharam destaque e passaram a ser considerados propósitos a serem alcançados, contemplados nos Objetivos do Desenvolvimento Sustentável, metas 5.1 e 5.2^4^.

O termo *violência obstétrica* foi construído por movimentos civis em defesa dos direitos reprodutivos das mulheres e incorporado à legislação da Venezuela em 2007. Desde então, a terminologia foi adotada por outros países e é amplamente utilizada e difundida em todo o mundo. Embora não exista consenso sobre sua nomenclatura nem definição^5^, de maneira geral, a violência obstétrica é caracterizada por abusos físicos, sexuais, psicológicos, negligência, intervenções médicas desnecessárias ou não baseadas em evidências científicas, falta de informação ou consentimento das mulheres para realização de procedimento, privação de direitos previstos em leis e restrições estruturais, de insumos e recursos humanos nos ambientes de saúde^5,6^. Assim, é consenso que sua ocorrência consiste em uma violação dos direitos humanos, incluindo o direito à saúde, à integridade física e à autonomia reprodutiva^2^.

A violência obstétrica tem algumas características específicas: ocorre durante o ciclo reprodutivo, abrangendo a gestação, parto, puerpério e abortamento; manifesta-se nos serviços de saúde, incluindo ambulatórios, consultórios e maternidades/hospitais; os perpetradores são, frequentemente, profissionais de saúde (embora não se restrinja a essa categoria); e provoca consequências negativas na saúde da mulher e do seu recém-nascido^7–9^.

No Brasil, a prevalência de violência obstétrica varia entre 18 e 44%, conforme dados de estudos nacionais^10^, seja de base hospitalar^8^, seja populacional^11^. A grande amplitude dessas estimativas decorre de diferentes fatores, como o local de realização da pesquisa, o período pós-parto em que foi realizada e a metodologia utilizada para o cálculo da prevalência (pontual ou ao longo da vida). Todavia, o uso de diferentes questionários e perguntas para a mensuração do problema representa uma das principais limitações, prejudicando tanto a precisão das estimativas quanto a comparabilidade dos dados.

Em 2021, a prefeitura do Rio de janeiro lançou um canal para denúncias para violência obstétrica por meio do número 1746^12^, no entanto denúncias de violência obstétrica têm sido frequentes em todo o estado. Elas ocorrem por telefone e outras vias (tais como a mídia e as redes sociais), culminando em casos emblemáticos, como o ocorrido em 2023, quando uma mulher foi estuprada por um anestesista durante uma cesariana, contudo não há pesquisas epidemiológicas representativas sobre violência obstétrica no estado, constituindo uma importante lacuna na literatura.

Com o intuito de solucionar algumas das limitações supracitadas, a Pesquisa Nascer no Brasil II teve como objetivos estimar a prevalência de violência obstétrica em mulheres internadas por motivos de parto no estado do Rio de Janeiro; examinar a prevalência em subgrupos vulneráveis; e investigar o perfil de coocorrência de diferentes tipos de violência obstétrica nessa população.

## MÉTODOS

### Desenho de Estudo

Trata-se de uma coorte perinatal de base hospitalar e representatividade nacional. A coorte foi composta de um *baseline* e dois seguimentos por telefone realizados no segundo e no quarto mês pós-parto ou aborto. O protocolo da pesquisa foi publicado anteriormente, em que informações detalhadas sobre o *baseline* ^13^ e o seguimento^14^ podem ser encontradas.

### População de Estudo

A população do estudo da Pesquisa Nascer no Brasil II é composta de todas as mulheres internadas por motivo de parto (de nascido vivo ou natimorto) e em situações de abortamento, em maternidades/hospitais selecionados em todo o Brasil com 100 ou mais nascidos vivos segundo o Sistema de Informações sobre Nascidos Vivos de 2017. Foram excluídas mulheres com gestação trigemelar ou mais, com distúrbios mentais graves, estrangeiras que não entendiam o português e surdas.

O presente estudo teve como critério de inclusão as mulheres do estado do Rio de Janeiro e mulheres internadas em virtude do parto. Foram excluídas mulheres cujos bebês foram natimortos e pesando menos de 500 gramas ou com idade gestacional menor que 20 semanas (abortos).

### Plano Amostral

A amostra de puérperas do estado do Rio de Janeiro foi planejada para representar seu território. Assim, 29 maternidades foram incluídas, 14 públicas, 12 privadas e três mistas. A amostra foi calculada com base na proporção de cesarianas no estado do Rio de Janeiro em 2019 (57%), nível de significância de 5% e potência de 90% para detectar diferenças de 7%. Utilizamos o efeito de desenho de 1,3, resultando em um tamanho amostral mínimo de 1.350 puérperas. Cálculos *post-hoc* indicam que a amostra tem poder de detectar diferenças absolutas de 5% para desfechos com prevalência entre 5 e 20%.

### Fonte de Dados

Este estudo utilizou a entrevista hospitalar com a mulher após o parto (informações sociodemográficas, contatos telefônicos, pré-natal e assistência hospitalar) e o questionário do segundo seguimento telefônico (violência obstétrica) como fontes de informação.

### Procedimento de Coleta de Dados

As mulheres foram abordadas em seu próprio leito hospitalar, em quartos individuais ou enfermarias coletivas, no mínimo seis horas após o parto, por uma entrevistadora treinada. Nessa oportunidade, elas foram convidadas a fazer parte da pesquisa, que consistiu em três entrevistas, sendo a primeira ainda no hospital (*baseline*) e dois seguimentos por telefone. Também foi pedida autorização para a coleta de informações do cartão de pré-natal e prontuário.

### Variáveis e Instrumentos de Mensuração

As variáveis demográficas, socioeconômicas e sobre a gestação atual foram extraídas do questionário hospitalar. No tocante às variáveis demográficas, a idade da mulher (em anos) foi classificada em três categorias: adolescentes (≤ 19 anos), adultas (20–34 anos) e adultas com maior risco obstétrico (35 anos ou mais). A cor da pele autorreferida foi coletada de acordo com o recomendado pelo Instituto Brasileiro de Geografia e Estatística em cinco categorias: branca, preta, parda, amarela e indígena. No entanto, no estudo atual, por causa da baixa autoidentificação de mulheres como indígenas (5) ou amarelas (3), a informação da cor dessas mulheres foi excluída da análise, configurando-se em três categorias: branca, preta e parda. A escolaridade da mulher foi aferida de acordo com o último grau escolar concluído e categorizada em até oito anos de estudo (correspondendo ao ensino fundamental incompleto), entre nove e 11 anos de estudo e maior ou igual a 12 anos de estudo (correspondendo ao ensino médio incompleto e ao ensino médio completo, respectivamente). A situação conjugal foi categorizada em vivendo com ou sem companheiro.

Em relação às características socioeconômicas, foi perguntado à mulher se ela teve trabalho remunerado ao longo da gestação (sim ou não); e se ela ou alguém do seu domicílio recebeu Bolsa Família ou outro benefício social do governo, incluindo o auxílio emergencial durante a pandemia de COVID-19 (sim ou não).

Em relação à gestação atual, a paridade da mulher foi categorizada em primípara ou multípara. Também foi perguntado sobre a adequação do pré-natal, sendo consideradas adequadas aquelas mulheres que fizeram oito ou mais consultas ao longo da gestação^15^. Além disso, foi perguntado à mulher se ela entrou em trabalho de parto no hospital/maternidade onde o bebê nasceu (sim ou não), a duração do trabalho de parto (< 2 horas, de 2 a 8 horas e ≥ 8 horas), a presença de acompanhante o tempo todo (sim ou não), a presença de doula no trabalho de parto ou parto (sim ou não), a via de parto (vaginal, incluindo fórceps ou vácuo, ou cesariana) e por fim, sobre a fonte de financiamento do parto (público ou privado). A altura e o peso antes da gestação foram informados pela mulher na entrevista hospitalar. Assim, o índice de massa corporal pré-gestacional foi calculado e categorizado em eutrofia, sobrepeso e obesidade.

O questionário sobre violência obstétrica foi aplicado durante o segundo seguimento telefônico, quatro meses após o parto. Foi utilizada uma versão adaptada transculturalmente do questionário proposto por Bohren et al.^6^. O instrumento contempla cinco dimensões: abuso físico (sete itens); abuso psicológico (13 itens); negligência (quatro itens); estigma e discriminação (6 itens); e toques vaginais inadequados (cinco itens), totalizando 35 itens.

Para estimar a prevalência por dimensão, foi considerada a presença de violência obstétrica a resposta positiva em pelo menos um item da referida dimensão. Para estimar a prevalência total de violência obstétrica, considerou-se a resposta positiva em pelo menos um item de alguma das cinco dimensões analisadas.

### Análise Estatística

Todas as análises foram conduzidas considerando os pesos amostrais do *baseline*, bem como os pesos amostrais dos seguimentos baseados em características hospitalares ou maternas que se mostraram associadas à resposta da segunda entrevista por telefone. Essa calibração foi utilizada no intuito de minimizar o efeito das perdas de seguimento. Dessa forma, foi ajustado um modelo de regressão logística para estimar a probabilidade de cada mulher responder à entrevista telefônica, utilizando um conjunto de variáveis que diferenciaram as respondentes das não respondentes, por meio de escore de propensão. Esse procedimento teve o intuito de compensar a tendência de as mulheres com certas características não responderem à entrevista telefônica, supondo que teriam fornecido, em média, respostas semelhantes das respondentes em cada estrato e categoria de ajustamento. Foram testadas variáveis relacionadas ao local do parto, sociodemográficas e obstétricas. O modelo logístico mais parcimonioso incluiu as variáveis estatisticamente significativas (p < 0,05) que diferenciaram respondentes de não respondentes. As não respondentes tinham menos de 16 anos de estudo, o parto ocorreu em hospitais públicos (em comparação com os hospitais privados e mistos) e naqueles com menos de 500 partos/ano. As principais causas de não resposta foram telefone errado e não atender às ligações.

Em seguida, foi realizada uma análise descritiva das características demográficas, socioeconômicas e referentes à gestação atual da população de estudo. Foram analisadas as prevalências dos diferentes tipos de violência obstétrica em subgrupos, conforme as características supracitadas. Utilizou-se o teste χ^2^ para avaliar a homogeneidade das prevalências entre os subgrupos populacionais, adotando-se o valor p < 0,05 para identificar diferenças estatisticamente significativas.

O perfil de coocorrência da violência obstétrica foi analisado de duas formas. A primeira abordagem envolveu a contagem do número de tipos de violência obstétrica sofrido, sendo as prevalências também analisadas em subgrupos. A segunda contemplou a representação gráfica da coocorrência por meio do diagrama de Venn, agrupando-se os quatro tipos de violência obstétricas mais frequentes.

Todas as análises foram realizadas no *software* Stata versão 17.0, com o auxílio do comando *svy*, de modo a considerar a estrutura de amostragem complexa.

### Aspectos Éticos

Este estudo foi aprovado pela Comissão Nacional de Ética em Pesquisa (parecer nº 3.909.299) e pelos comitês de ética em pesquisa locais, sempre que exigido pelos hospitais selecionados. Antes de cada entrevista, foi solicitada a concordância da puérpera após a leitura do Termo de Consentimento Livre e Esclarecido. Para menores de idade, foi utilizado o termo de assentimento.

## RESULTADOS

Foram entrevistadas 1.923 puérperas na linha de base da pesquisa. Foram excluídos 120 casos de aborto, totalizando 1.881 puérperas. O segundo seguimento telefônico entrevistou 1.190 mulheres, representando 67,5% da amostra do *baseline*. A maior parte das entrevistadas (72,7%; intervalo de confiança de 95% — IC95% 69,8–75,3) tinha entre 20 e 34 anos. A maioria autodeclarou-se parda (44,4%; IC95% 39,3–49,6) e tinha ensino médio ou superior completo (56,6%; IC95% 45,7–66,9). Em relação à situação conjugal, 88,6% (IC95% 86,3–90,5) delas tinham companheiro; 52,5% (IC95% 48,9–56,2) não trabalharam durante toda a gestação; e 57,3% (IC95% 34,5–51,4) receberam algum benefício social do governo, tais como Bolsa Família e/ou Auxílio Brasil. Em relação ao estado nutricional pré-gestacional, 30,7% (IC95% 26,2–35,6) das mulheres tinham sobrepeso e 25,5% (IC95% 21,4–35,6) obesidade. A maioria das mulheres era multípara (63,2%; IC95% 60,5–65,9) e realizou o pré-natal de forma adequada (78,9%; IC95% 75,7–81,7) (dados não mostrados em tabela).

No que tange ao período periparto e parto, 44,8% (IC95% 38,5–51,2) das mulheres entraram em trabalho de parto, sendo a duração do trabalho de parto entre duas e oito horas para a maior parte delas (43,3%; IC95% 39,3–47,3). Quase a totalidade das mulheres teve acompanhante durante todo o tempo (95,0%; IC95% 85,4–98,4), entretanto só uma pequena parcela das mulheres contou com a presença de doula durante o trabalho de parto ou parto (2,9%; IC95% 1,9–4,4). A maior parte das mulheres fez cesariana (59,8%; IC95% 52,1–67,0), e, por fim, a maioria dos nascimentos ocorreu por meio de financiamento público (73,7%; IC95% 60,3–83,8) (dados não mostrados em tabela).

A Tabela 1 apresenta a frequência de cada ato de violência sofrido, bem como a prevalência por tipo de violência obstétrica e total. Os tipos de violência obstétrica mais prevalentes foram os toques vaginais inadequados (46,2%), seguidos de negligência (31,5%), abuso psicológico (21,7%), estigma e discriminação (7,8%) e abuso físico (3,1%). A prevalência de violência obstétrica total foi de 65,3%. No tocante ao perpetrador das violências obstétricas sofridas, o profissional da enfermagem foi o profissional mais citado pelas mulheres entrevistadas. Em relação ao momento de ocorrência da violência obstétrica, a maioria ocorreu no período periparto/parto.

**Tabela 1:** Prevalência segundo ato de violência sofrido, natureza da violência e total em puérperas residentes no Estado do Rio de Janeiro, RJ, 2022/2023, Estudo Nascer no Brasil 2 (n = 1.761).

		Amostra total		
		n	%	IC95%
**Atos de abuso físico sofridos**				
	Beliscar	-	-	-
	Levar tapa ou soco	-	-	-
	Levar chute	-	-	-
	Bater com algum objeto	1	0,08	0,01–6,62
	Amordaçar ou colocar a mão na boca para impedir de falar ou fazer barulho	-	-	-
	Segurar à força, enforcar ou amarrar na cama	4	0,38	0,11–1,28
	Apertar a barriga ou subir nela para "ajudar" a saída do bebê	53	2,68	2,00–3,61
	Ter sofrido pelo menos um ato de abuso físico	58	3,14	2,39–4,12
**Categoria profissional do perpetrador do abuso físico** a				
	Médico/Médica	1	21,73	0,09–98,8
	Enfermagem	2	45,64	19,67–85,50
	Outros profissionais	1	15,71	0,23–93,87
	Desconhecido	1	16,92	0,06–98,45
**Em qual momento o abuso físico aconteceu**				
	Pré-parto/parto	11	65,59	47,50–80,07
	Pós-parto	5	29,71	15,55–49,23
	Ambos os momentos	2	4,70	0,41–37,17
**Atos de abuso psicológico sofridos**				
	Gritar ou berrar	49	4,61	3,68–5,76
	Ofender ou xingar	41	3,12	1,71–5,63
	Repreender ou dar bronca	121	10,87	9,34–12,63
	Debochar	85	6,85	4,57–10,15
	Comentários negativos sobre sua aparência física	18	1,46	0,63–3,32
	Comentários negativos sobre a aparência física do bebê	6	0,47	0,19–1,17
	Comentários negativos sobre vida sexual	39	3,70	2,83–4,84
	Ameaçar de fazer algum procedimento médico que você não queria	37	3,78	2,57–5,53
	Ameaçar de abuso físico	1	0,13	0,02–1,07
	Ameaçar dizendo que, se não obedecer, mulher e bebê teriam problemas	32	2,96	2,28–3,82
	Ameaçar não cuidar ou parar de cuidar da mulher ou do bebê	14	1,18	0,07–2,06
	Culpar mulher por alguma coisa que aconteceu com ela ou bebê	40	3,93	2,97–5,19
	Bufar ou resmungar	106	10,10	8,59–11,84
	Ter sofrido pelo menos um ato de abuso psicológico	209	21,68	17,16–27,00
**Categoria profissional do perpetrador do abuso psicológico**				
	Médico/Médica	52	23,31	18,76–28,57
	Enfermagem	108	54,35	46,37–62,12
	Outros profissionais	25	10,90	6,07–18,81
	Desconhecido	21	11,44	8,31–15,54
**Em qual momento o abuso psicológico aconteceu**				
	Pré-parto/parto	102	48,59	38,26–59,03
	Pós-parto	77	38,97	27,17–52,22
	Ambos os momentos	25	12,45	6,78–21,75
**Atos de negligência sofridos**				
	Sentir-se ignorada pelos profissionais de saúde e outros funcionários	207	19,95	17,63–22,49
	Sentir-se abandonada pelos profissionais de saúde e outros funcionários	168	17,01	14,14–20,32
	Sentir que sua presença era um incômodo	84	8,29	6,92–9,89
	Precisar esperar por longos períodos de tempo antes de ser atendida	250	22,2	19,21–25,50
	Ter sofrido pelo menos um ato de negligência	908	31,45	29,11–33,89
**Atos relacionados à estigma e discriminação sofridos**				
	Receber comentários negativos por causa da sua raça/cor da pele	2	0,48	0,13–1,75
	Receber comentários negativos por causa da sua religião	5	0,37	0,12–1,09
	Receber comentários negativos por causa da sua idade	56	4,78	3,68–6,18
	Receber comentários negativos sobre ter/não ter companheiro	18	1,81	1,05–3,09
	Receber comentários negativos por causa da sua escolaridade ou situação financeira	6	0,39	0,14–1,08
	Receber comentários negativos por causa da sua condição de saúde	18	1,77	0,70–4,40
	Ter sofrido pelo menos um ato de estigma e discriminação	81	7,79	6,25–9,66
**Categoria profissional do perpetrador do estigma e discriminação**				
	Médico/Médica	14	19,99	13,22–29,07
	Enfermagem	38	43,58	32,63–55,20
	Outros profissionais	11	16,06	9,87–24,99
	Desconhecido	18	20,37	14,22–28,30
**Em qual momento o ato de estigma e discriminação aconteceu**				
	Pré-parto/parto	32	45,16	32,23–58,77
	Pós-parto	23	32,80	22,09–45,67
	Ambos os momentos	14	22,04	9,87–42,20
**Atos relacionados aos toques vaginais inadequados**				
	Não explicou por que o exame vaginal era necessário	363	35,24	29,50–41,45
	Não pediu sua permissão antes de fazer o exame vaginal	226	22,48	17,66–28,18
	Falar informações confidenciais sobre a sua saúde de forma que outras pessoas pudessem ouvir	56	5,34	3,78–7,51
	Os exames vaginais não foram feitos de forma privada/ com privacidade	221	19,81	16,60–23,47
**Ter sofrido pelo menos um toque vaginal inadequado**		435	46,23	39,46–53,15
**Ter sofrido pelo menos um tipo de violência obstétrica**		1.219	65,30	58,60–71,44

IC95%: intervalo de confiança de 95%.

a A pergunta referente ao perpetrador do ato de abuso físico foi perguntada na ligação telefônica 2, na qual não foi realizada a pergunta referente à ocorrência de Kristeller (i.e., apertar a barriga ou subir nela para "ajudar" a saída do bebê). Essa questão estava inserida no questionário hospitalar (linha de base).

A Tabela 2 apresenta as prevalências dos tipos de violência de acordo com as características das mulheres entrevistadas. A prevalência de violência obstétrica, considerando todas as dimensões, foi maior entre as mulheres sem trabalho remunerado e com o parto com financiamento público. Mulheres que recebiam algum auxílio governamental também tiveram maior prevalência de violência obstétrica considerando todas as dimensões, exceto abuso físico. A idade acima de 35 anos, a escolaridade inferior a 12 anos de estudo e a presença de trabalho de parto foram características marcadoras da violência obstétrica em três diferentes manifestações (negligência; estigma e discriminação; e toques vaginais inadequados). Mulheres primíparas e que tiveram o trabalho de parto superior a oito horas também apresentaram maiores prevalências de violência obstétrica em duas diferentes dimensões (abuso psicológico e negligência). Por fim, mulheres pretas, sem companheiro e com o pré-natal inadequado apresentaram maior prevalência de estigma e discriminação.

**Tabela 2:** Prevalência de violência obstétrica por tipologia de acordo com características demográficas, socioeconômicas e referentes à gestação atual de puérperas residentes no Estado do Rio de Janeiro, RJ, 2022/2023, Estudo Nascer no Brasil 2 (n = 1.761).

		Abuso físico		Abuso psicológico		Negligência		Estigma e discriminação		Toques vaginais inadequados	
		%	IC95%	%	IC95%	%	IC95%	%	IC95%	%	IC95%
**Idade (anos)**											
	**≤** 19	3,13	2,35–4,16^a^	24,40	16,88–33,90	32,28	28,82–35,95	7,89	5,99–10,33^b^	45,97	40,16–51,90^a^
	20–34	1,21	0,26–5,38	8,65	4,62–15,64	26,87	20,57–34,26	2,01	0,83–4,77	40,23	30,11–51,25
	**≥** 35	6,46	3,59–11,37	24,01	11,82–42,71	33,17	20,30–49,17	16,54	11,30–23,56	58,99	47,66–69,45
**Raça/cor da pele**											
	Branca	3,75	2,39–5,84	18,44	10,71–29,89	30,07	25,51–35,06	5,61	3,48–8,96^a^	39,43	28,27–51,81
	Preta	0,85	0,18–4,05	24,75	18,03–32,98	29,25	25,03–33,86	10,95	8,61–13,82	51,73	39,66–63,60
	Parda	4,05	2,29–7,05	22,07	18,75– 25,79	33,63	29,53–38,00	7,44	5,38–10,21	47,45	42,36–52,61
**Escolaridade (anos de estudo)**											
	> 12	2,11	1,30–3,42	18,57	14,54–23,40^a^	29,44	23,23–32,86	4,78	3,21–7,05^a^	37,69	32,98–42,65^a^
	8–12	4,52	3,26–6,25	26,95	23,03–31,27	34,25	25,22–44,59	10,56	7,35–14,95	58,72	50,32–66,64
	< 8	4,43	1,64–11,41	22,86	15,24–32,81	33,86	25,62–43,20	14,19	7,90–24,15	53,67	44,18–62,91
**Situação conjugal**											
	Sem companheiro	5,39	2,85–9,97	22,42	16,90–29,12	37,86	30,56–45,75	14,61	9,31–22,18^a^	43,46	34,43–53,14
	Com companheiro	2,86	2,14–3,82	21,49	16,67–27,25	30,56	28,19–33,04	6,92	5,33–8,95	46,46	39,40–53,66
**Trabalho remunerado durante a gestação**											
	Não	4,25	3,09–5,83^a^	24,24	19,25–30,03^a^	33,84	31,19–36,61^a^	10,72	8,78–13,02^b^	53,45	43,27–63,35^a^
	Sim	1,92	1,20–3,04	18,85	14,21–24,57	28,80	25,31–32,57	4,55	3,07–6,69	38,21	33,23–43,45
**Recebe(eu) Bolsa Família, benefício social ou auxílio emergencial do governo**											
	Não	3,09	1,61–5,86	16,21	11,90–21,68^a^	25,94	22,73–29,42^b^	3,61	2,02–6,38^a^	40,70	32,77–49,14^a^
	Sim	3,19	2,25–4,51	25,85	20,26–32,35	35,59	32,26–39,08	10,91	8,69–13,61	50,13	44,05–56,20
**Paridade**											
	Primípara	4,57	3,14–6,61^a^	26,49	20,43–33,60^b^	35,15	30,12–40,53	7,77	5,19–11,49	45,54	41,28–49,87
	Multípara	2,32	1,44–3,71	18,8	14,89–23,42	29,33	25,62–33,34	7,81	5,99–10,12	46,68	37,79–55,78
**Adequação ao pré-natal**											
	Não	6,89	4,28–10,89	21,79	12,73–34,75	37,15	29,70–45,26	15,29	11,21–20,52^b^	50,76	46,20–55,31
	Sim	2,32	1,52–3,53^a^	21,92	14,60–31,57	30,41	27,86–33,09	5,83	4,41–7,66	44,89	36,90–53,15
**Entrou em trabalho de parto**											
	Não	0,83	0,22–3,03^a^	19,01	12,05–28,67	27,06	22,57–32,08^a^	7,06	5,08–9,74	42,67	36,14–49,45^a^
	Sim	6,08	4,13–8,85	24,93	21,57–28,64	36,93	33,14–40,90	8,62	6,50–11,34	48,88	41,44–56,36
**Tempo de trabalho de parto (horas)**											
	< 2	0,00 -	20,37	12,33–31,75^a^	29,39	23,70–35,81^b^	9,13	5,45–14,92	50,02	38,55–61,48
	2 a 8	3,57 1,54–8,03	15,71	12,46–19,61	22,44	18,49–26,96	5,80	3,99–8,36	42,06	32,99–51,70
	**≥** 8	4,37 2,12–8,80	27,60	24,82–30,57	40,79	28,41–43,50	8,60	6,40–11,45	50,43	43,84–57,01
**Via de parto**											
	Vaginal	7,11 5,55–9,06^b^	23,70	21,14–26,47	33,18	27,70–39,17	7,42	5,38–10,17	50,68	40,92–60,40
	Cesariana	0,48 0,14–1,64	20,32	13,46–29,48	30,29	25,53–35,51	8,04	5,63–11,36	42,88	37,54–48,40
**Presença de acompanhante o tempo todo**											
	Não	3,77 1,11–12,08 18,82		5,59–47,56	35,67	28,91–42,76	6,96	5,34–9,02	56,44	43,92–68,20
	Sim	2,63 1,88–3,65	21,05	15,65–27,69	30,67	28,48–32,94	7,84	2,86–19,75	44,44	35,86–53,86
**Presença de doula**											
	Não	2,79 1,96–3,97	21,55	16,41–27,75	32,26	29,33–35,33	7,65	5,90–9,86	45,21	39,29–51,27
	Sim	11,14 2,62–36,86	26,32	14,39–43,17	13,56	4,51–34,25	2,33	0,30–15,97	56,36	30,75–78,98
**Fonte de financiamento do parto**											
	Público	4,04 3,08–5,26^b^	26,66	23,01–30,65^b^	35,03	32,57–38,13^b^	10,06^a^	8,36–12,05^b^	51,39	45,02–57,72^b^
	Privado	0,65 0,21–2,00	7,43	4,00–13,38	20,64	16,08–26,09	1,33	0,55–3,16	29,39	22,82–36,96
**IMC pré-gestacional**											
	Eutrofia	3,08 1,55–6,02	23,83	13,69–38,15	34,60	27,45–42,51	7,75	5,64–10,56	44,51	32,53–57,16
	Sobrepeso	3,62 1,79–7,17	16,01	11,04–22,64	27,75	23,06–32,98	5,02	2,50–9,82	42,44	32,71–52,80
	Obesidade	1,92 0,47–7,52	22,01	17,08–27,89	31,85	26,34–37,91	7,04	4,36–11,18	43,17	33,73–53,13

IC95%: Intervalo de confiança de 95%; IMC: índice de massa corporal.

a p ≤ 0.05;

b p ≤ 0.01.

O acúmulo de diferentes formas de violência obstétrica sofrida, de acordo com algumas características das mulheres entrevistadas, está apresentado na Tabela 3. Observa-se que cerca de 1/3 das puérperas relataram ter vivenciado pelo menos um tipo de violência obstétrica. Destaca-se que puérperas com mais de 35 anos e as adolescentes, mulheres com menos de 12 anos de escolaridade, sem trabalho e/ou que receberam benefício do governo, primíparas, que entraram em trabalho de parto, com trabalho de parto prologado, com parto vaginal e que tiveram assistência ao parto com financiamento público vivenciaram mais formas de violência obstétrica, quando comparadas às mulheres sem essas características.

**Tabela 3:** Acúmulo de diferentes formas de violência obstétrica sofrida segundo características demográficas, socioeconômicas e referentes à gestação atual de puérperas residentes no estado do Rio de Janeiro, RJ, 2022/2023, Estudo Nascer no Brasil 2 (n = 1.761).

		0 VO		1 VO		2 VO		+3 VO		p-valor
		%	IC95%	%	IC95%	%	IC95%	%	IC95%	
**Amostra total**		38,03	30,51–46,18	32,56	28,44–36,96	16,64	13,73–20,01	12,77	10,43–15,54	
**Idade (anos)**										
	**≤** 19	39,10	32,41–46,23	27,82	24,50–31,41	19,02	14,71–24,22	14,06	10,31–18,88	0,0204
	20–34	43,57	29,41–58,86	45,42	27,86–64,21	6,26	2,89–13,03	4,75	2,26–9.68	
	**≥** 35	19,60	13,43–27,68	45,54	27,84–64,44	17,29	10,40–27,35	17,57	9,85–29,37	
**Raça/cor da pele** a										
	Branca	42,37	29,89–55,92	32,40	25,17–40,58	15,14	8,92–24,55	10,08	7,69–13,11	0,3157
	Preta	35,94	28,48–44,15	33,91	28,62–39,65	14,45	10,90–18,90	15,70	10,37–23,06	
	Parda	36,41	30,67–42,56	31,87	27,24–36,88	18,90	16,11–22,04	12,83	9,82–16,59	
**Escolaridade (anos de estudo)**										
	> 12	44,22	37,00–51,69	33,84	7,64–40,64	12,50	10,21–15,21	9,45	7,45–11,91	0,0004
	8–12	28,06	22,34–34,6	29,18	24,21–34,71	24,40	19,95–29,47	18,35	14,77–22,58	
	< 8	34,97	23,98–47,82	34,33	27,03–42,45	16,58	10,55–25,08	14,12	6,73–27,26	
**Situação conjugal**										
	Sem companheiro	38,84	31,36–46,88	32,85	28,74–37,24	15,89	13,02–19,26	12,43	9,79–15,65	0,1466
	Com companheiro	32,51	21,88–45,32	30,33	20,9–41,77	22,70	17,73–28,57	14,46	10,19–20,11	
**Trabalho remunerado durante a gestação**										
	Não	30,67	19,68–44,41	36,83	27,41–47,38	16,78	14,57–19,25	15,72	12,16–20,08	0,0654
	Sim	46,07	41,52–50,69	27,89	22,17–34,45	16,48	10,81–24,32	9,55	7,80–11,66	
**Recebe(eu) Bolsa Família, outro benefício social ou auxílio emergencial do governo**										
	Não	43,82	35,24–52,78	33,41	27,03–40,47	13,72	9,00–20,36	9,05	6,48–12,51	0,0232
	Sim	33,92	26,78–41,87	31,87	27,39–36,71	18,76	16,31–21,47	15,46	11,57–20,35	
**Paridade**										
	Primípara	37,53	30,10–45,61	27,29	22,09–33,19	16,41	12,48–21,28	18,77	13,60–25,32	0,0046
	Multípara	38,36	29,88–47,62	35,47	29,12–42,37	16,78	13,68–20,42	9,39	8,08–10,88	
**Adequação ao pré-natal**										
	Sim	33,15	27,52–39,3	31,91	25,21–39,45	23,05	18,36–28,51	11,89	6,75–20,12	0,1951
	Não	39,03	29,91–48,99	32,84	28,31–37,72	14,80	11,23–19,27	13,33	10,16–17,30	
**Entrou em trabalho de parto**										
	Não	43,36	34,95–52,16	32,99	27,95–38,47	12,88	9,38–17,43	10,77	6,78–16,68	0,0153
	Sim	32,99	25,91–40,93	31,38	27,92–35,07	20,63	16,71–25,20	14,99	12,05–18,51	
**Tempo de trabalho de parto (horas)**										
	< 2	36,37	23,85–51,06	33,24	26,57–40,66	17,18	12,55–23,07	13,2	9,51–18,05	0,0077
	2 a 8	44,41	34,55–54,74	35,19	34,55–54,74	14,41	8,56–23,24	5,99	3,94–9,01	
	**≥** 8	32,48	28,08–37,22	28,63	24,36–33,33	19,93	15,52–25,24	18,95	15,29–23,24	
**Via de parto**										
	Vaginal	33,72	27,85–40,14	32,00	27,32–37,07	22,57	19,28–26,24	11,71	9,27–14,67	0,0149
	Cesariana	41,28	32,19–51,01	32,97	28,34–37,96	12,18	8,73–16,75	13,57	9,11–19,72	
**Presença de acompanhante o tempo todo**										
	Não	38,12	28,40–48,90	26,22	15,21–41,33	19,08	10,90–31,25	16,57	5,86–38,82	0,6822
	Sim	38,80	30,01–48,37	32,57	28,03–37,46	16,81	13,88–20,22	11,82	8,77–15,73	
**Presença de doula**										
	Não	38,86	31,43–46,86	31,87	27,76–36,29	16,05	13,30–19,25	13,21	10,33–16,74	0,3773
	Sim	38,40	18,36–63,33	22,08	8,14–47,53	31,05	8,73–67,94	8,48	2,31–26,63	
**Fonte de financiamento do parto**										
	Público	32,55	25,66–40,28	31,83	27,20–36,86	20,06	17,89–22,42	15,56	13,63–17,71	0,0000
	Privado	55,78	47,39–63,86	34,90	25,14–46,12	5,58	3,07–9,94	3,74	1,73–7,88	
**Índice de massa corporal pré-gestacional**										
	Eutrofia	39,77	27,81–53,10	26,56	22,28–31,33	19,26	12,88–27,77	14,41	8,44–23,52	0,2972
	Sobrepeso	41,37	33,37–49,85	36,55	28,32–45,65	11,69	7,01–18,85	10,39	6,66–15,86	
	Obesidade	43,85	33,49–54,77	28,28	17,98–41,49	12,16	7,69–18,70	15,71	10,80–22,31	

VO: Violência obstétrica; IC95%: intervalo de confiança de 95%.

O perfil de coocorrência de violência obstétrica em puérperas residentes no estado do Rio de Janeiro é apresentado graficamente na Figura 1. Observa-se que 38% da amostra relatou não ter sido vítima de pelo menos um dos tipos de violência obstétrica mais prevalentes no estado. A coocorrência dos quatro tipos de violência obstétrica mais prevalentes (abuso psicológico, negligência, estigma e discriminação e toques vaginais inadequados) foi de 3%.

**Figura 1:** Diagrama de Veen: puérperas do estado do Rio de Janeiro (ERJ).

## DISCUSSÃO

Os achados do presente estudo mostram que a violência obstétrica é um fenômeno frequente nas maternidades do estado do Rio de Janeiro. Aproximadamente 2/3 das mulheres entrevistadas relataram ter sofrido pelo menos um tipo das violências investigadas, sendo os toques vaginais inadequados e a negligência as formas mais comuns. Observou-se que os períodos do pré-parto e do parto foram os momentos de maior ocorrência da violência obstétrica e que os profissionais de enfermagem foram os principais perpetradores. Os resultados também indicaram desigualdade na ocorrência desse problema, com maior vulnerabilidade entre mulheres em condições socioeconômicas e demográficas desfavoráveis. Além disso, algumas características fisiológicas do parto foram marcadoras da ocorrência de violência obstétrica.

Comparando nossos achados com os resultados do estudo de Bohren et al.^16^, um estudo multicêntrico realizado em quatro países africanos (Gana, Guiné, Myanmar e Nigéria) mostrou prevalência de toques vaginais sem consentimento de 59,4%, valor superior ao encontrado no presente estudo, enquanto negligência foi reportada por 4,5% das mulheres, estimativas bem aquém das encontradas nesta pesquisa. Para os demais tipos de violência (abuso físico, psicológico, estigma e discriminação), Bohren et al.^16^ encontraram prevalência média de 14, 37,8 e 0,6%, respectivamente, com valores quatro vezes maiores de abuso físico em países africanos e maiores frequências de violência psicológica e estigma e discriminação no estado do Rio de Janeiro, sendo a discriminação por idade a mais frequente. Tais discrepâncias nos achados podem ser explicadas pelas diferenças culturais e socioeconômicas, serviço de saúde local, modelo de gestão em saúde vigente, bem como percepção de violência obstétrica por parte das mulheres entrevistadas nesses locais.

Em relação à prevalência segundo as características demográficas e socioeconômicas, ressalta-se que a vivência de atos de violência obstétrica foi mais relatada por mulheres que apresentavam características como ser adolescente ou ter mais de 35 anos; ausência de trabalho remunerado durante a gestação, recebimento de auxílio governamental, parto com financiamento público. Mulheres nessas condições apresentaram maior prevalência de quase todos os tipos de violência obstétrica, com exceção do abuso físico. O mesmo padrão foi observado no acúmulo de diferentes experiências de violência. De maneira geral, ter sofrido três ou mais formas de violência obstétrica foi mais comum entre mulheres em pior situação socioeconômica. Esses achados mostram que violência obstétrica não ocorre de forma aleatória, mas é direcionada às mulheres mais pobres, evidenciando as desigualdades na prestação dos serviços de saúde e ferindo o princípio de igualdade garantido pela Constituição Federal brasileira^17^ e reforçada pela Convenção de Belém do Pará^18^.

Em relação às características da gestação atual, ser primípara, ter entrado em trabalho de parto e ter um trabalho de parto prolongado foram os principais marcadores da violência obstétrica. Esse resultado evidencia o despreparo das equipes de saúde no acompanhamento do trabalho de parto e no fornecimento de suporte emocional, sendo, ao contrário, causadores de violência obstétrica. Corroborando esses resultados, também foi observado que todos os tipos de violência obstétrica têm o período pré-parto e parto como os principais momentos de sua ocorrência^16^. Nesses períodos, a interação da mulher com a equipe de saúde é mais intensa, além de ser o momento de maior tensão para todos os envolvidos. Esses achados são semelhantes ao que é observado em outros países do mundo, conforme mostrado na pesquisa de Bohren et al.^16^.

Outra característica comum a todas as formas de violência obstétrica analisadas foi o perpetrador. As mulheres entrevistadas apontaram o profissional de enfermagem como o principal responsável pela maioria dos atos sofridos. Embora pareça paradoxal, considerando que a enfermagem é uma das principais defensoras do movimento de humanização do parto, outros estudos na América Latina e em outras regiões do mundo já haviam mostrado resultados semelhantes^16,19^. Isso pode ser explicado pelo fato de essa categoria profissional ser responsável pelo cuidado longitudinal, passando mais tempo em contato próximo com essas mulheres, especialmente durante o trabalho de parto, que é o momento mais tenso e em que mais ocorrem situações de violência obstétrica. Condições precárias de trabalho, como equipes desfalcadas e sobrecarga de trabalho, podem causar o esgotamento físico e emocional da equipe, contribuindo para as violências mais relatadas pelas mulheres, como "bufar e resmungar", "repreender ou dar bronca" e "sentir-se ignorada ou abandonada pelos profissionais". Deve-se destacar, também, que a categoria enfermagem inclui profissionais de níveis médio e superior. Assim, a mitigação da violência obstétrica perpassa pela garantia de adequadas condições de trabalho e pela capacitação e valorização do profissional de enfermagem.

Algumas particularidades foram observadas a depender dos tipos de violência obstétrica investigados. Em relação à violência física, sua prevalência foi pequena, sendo impulsionada principalmente pela manobra de Kristeller. No entanto, de acordo com o Sistema de Informações sobre Nascidos Vivos, aproximadamente 180 mil crianças nasceram em 2022 no estado do Rio de janeiro. Projetando a prevalência encontrada (3,14%), isso significa que cerca de 5.600 mulheres sofreram uma das formas mais graves de violência física durante o parto no estado. Além disso, esse achado evidenciou que mulheres que tiveram parto vaginal com financiamento público representaram quase a totalidade dos casos de violência física. O mesmo padrão foi observado para estigma e discriminação, em que partos com financiamento público responderam por quase todos os casos. Uma possível explicação para esses resultados é o modelo de atenção adotado em partos com financiamento privado, com assistência pré-natal e ao parto prestada pelo mesmo profissional, o que explicaria menor frequência de estigma e discriminação. Também se verificou maior frequência de nascimentos por cesariana em mulheres com financiamento privado, o que explicaria a menor ocorrência da manobra de Kristeller. Ressalta-se, entretanto, que, mesmo em mulheres com partos com financiamento privado, a prevalência de toques vaginais inadequados e negligência foi superior a 20%. Ou seja, mulheres com parto com financiamento público são mais atingidas pela violência obstétrica, mas mulheres com parto com financiamento privado também são vitimadas.

Os resultados apresentados devem ser vistos à luz de seus pontos fortes e limitações. Como ponto forte, destaca-se a amostra representativa do estado do Rio de Janeiro, uma vez que não há estudo com representatividade estadual sobre violência obstétrica no país. Adicionalmente, é importante salientar que a utilização de um questionário específico para a mensuração de violência obstétrica endossado pela OMS não só evidencia a originalidade e qualidade metodológica da pesquisa, mas também permite maior comparabilidade com outros estudos.

Quanto às limitações, é necessário reconhecer a perda de seguimento de 32,5% em relação ao *baseline*, um valor semelhante ao observado em outras coortes perinatais^20^. Em análises prévias, foi constatado que essas perdas não foram aleatórias. Assim, foi utilizada uma correção estatística a qual minimizou a subestimação de violência obstétrica nessa população. Além disso, a classificação genérica da equipe de enfermagem impossibilita distinguir os profissionais de diferentes formações. Isso aconteceu, pois, em muitos casos, a mulher não reconhece a diferença entre eles.

Por fim, concluímos que a violência obstétrica é frequente entre as mulheres no estado do Rio de Janeiro. Características socioeconômicas desfavoráveis e fatores relacionados à gestação e ao parto são marcadores importantes de sua ocorrência, evidenciando a desigualdade no atendimento prestado às parturientes desse grupo. Medidas educativas sobre violência obstétrica voltadas para profissionais de saúde e também para as mulheres podem ser implementadas em hospitais/maternidades para mitigar esse problema.

Ainda assim, esperamos que esta pesquisa possa conscientizar os gestores e profissionais de saúde sobre a violência obstétrica e fomentar a elaboração de políticas públicas para reduzir sua ocorrência entre as parturientes do estado do Rio de Janeiro, oferecendo respeito e dignidade nesse momento tão importante e único da vida das mulheres.

## Data Availability

Os conjuntos de dados gerados e/ou analisados durante o estudo estão disponíveis com o autor correspondente mediante solicitação.
